# Postpartum Regression of a Presumed Cavernous Meningioma

**DOI:** 10.1155/2016/2649426

**Published:** 2016-03-15

**Authors:** See Yung Phang, Peter Whitfield

**Affiliations:** Department of Neurosurgery, Derriford Hospital, Plymouth PL6 8DH, UK

## Abstract

Meningiomas are known to be more common in females than males. They are also known in rare cases to grow in size during pregnancy, which can complicate its management. We describe a 31-year-old Caucasian woman who presented with blurring of her vision and diplopia during the third trimester of her pregnancy. Magnetic resonance imaging (MRI) showed a small left cavernous sinus meningioma. The patient was treated conservatively until her uncomplicated delivery. A postpartum MRI scan showed complete regression of the suspected meningioma. Currently the patient is contemplating a further pregnancy.

## 1. Introduction

Meningiomas are known to be more common in females than males and rarely present (1.6 in 100,000) [1] during pregnancy. However due to the hormonal state of the mother and the presence of the foetus, the management of such patients can be complex. Some meningiomas are known to be hormone-sensitive as evidenced by the following possible reasons: (1) meningiomas are three times more common in females than males when they are in their reproductive years; (2) there is an association between meningiomas and breast cancer; (3) the use of exogenous female sex steroids has been shown to increase the risk of meningiomas; and (4) a large majority of meningiomas express progesterone receptors [2]. In this paper we report a 31-year-old patient with clinical signs of meningioma that has regressed after delivery and poses difficult questions regarding advice about future pregnancies.

## 2. Case Presentation

A 31-year-old lady was referred to us by our local ophthalmology department because of a left sided 6th nerve palsy with no papilledema or optic atrophy. She initially experienced blurring of her vision and subsequently developed horizontal diplopia at 37-week gestation.

**Figure 1:**
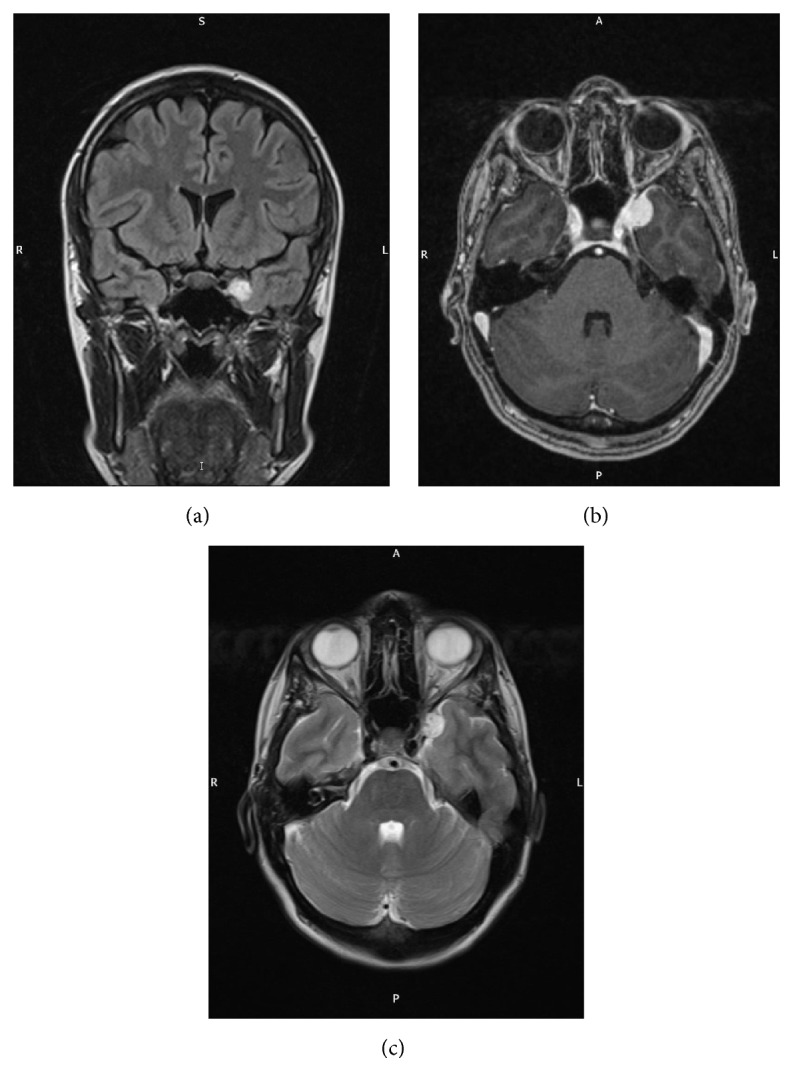
MRI images of a left sided lateral wall cavernous sinus meningioma coronal flair (a), T1 with contrast (b), and T2 axial (c).

**Figure 2:**
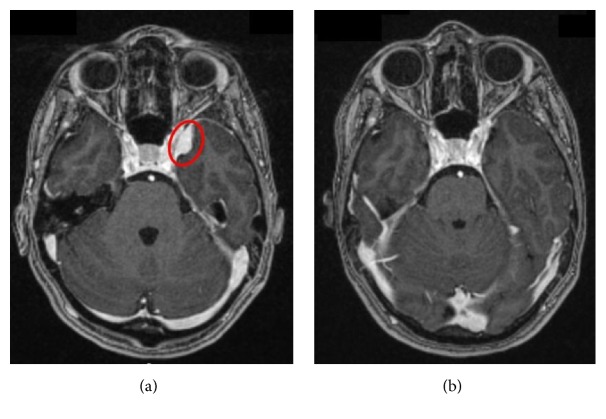
MRI illustrating the patient's meningioma (red): (a) 9 days before induction and (b) 8 months postpartum.

On neurological examination, a 6th nerve palsy was evident with no other focal neurological deficits present. Magnetic resonance imaging (MRI) showed a small left sided lateral wall cavernous sinus meningioma with some extension into the temporal fossa (Figure 1). The patient was managed conservatively with a view to possible treatment after delivery. She was induced at term and had a noncomplicated caesarean delivery. Some weeks after her pregnancy her symptoms started to improve and within a few months her vision was back to normal. Further MRI scans showed regression of the meningioma and the latest scan performed in one year after delivery showed no sign of any lesion (Figure 2). Currently, however, the patient is contemplating further pregnancy but is understandably worried about the risk of developing symptoms if the meningiomas were to reappear.

## 3. Discussion

Meningiomas have previously been reported to grow during pregnancy and regress postnatally [2]. The mechanism underlying this process has been postulated to be secondary to the hormonal effects of progesterone. When corrected for age and sex, pregnant patients with meningiomas have a larger portion of their tumours arising from the skull base than nonpregnant patients. This may be due to their close proximity to the cranial nerves leading to earlier presentation [2]. Symptoms including cranial nerve palsy, seizures, headaches, and vomiting warrant careful clinical assessment to avoid misdiagnosis as pregnancy related vitamin deficiencies, gestational diabetes, hyperemesis gravidarum, or preeclampsia. MRI with the lack of ionizing radiation is the radiological investigation of choice, where possible treatment is ideally deferred until postpartum. Conservative measures such as the use of steroids and anticonvulsants can help manage the patient's symptoms and buy valuable time until the foetus is delivered. Despite being an essential armamentarium in managing brain tumours, long-term use of steroids and anticonvulsants is known to cause hypoadrenalism and to be teratogenic, respectively. However the benefit of reducing intracranial pressure and preventing maternal and foetal hypoxia may outweigh these risks [1]. Caesarean delivery is preferred in cases where there are signs of raised intracranial pressure or where the patient has experienced seizures. Surgical resection of the tumour during pregnancy, although not unheard of, should be limited to patients with rapidly deteriorating neurology or impending herniation and where timely delivery of the foetus is not possible.

After delivery, a neurologically stable patient should be followed up for any clinical and radiological improvements or deterioration. Large, vascular meningiomas that are not treated immediately are at risk of swelling due to intravascular volume shifts in the patient after birth [3]. Other reasons advocating the need of resection of a meningioma are (1) the potential risk of progressive tumour growth secondary to progesterone secretion in the luteal phase of the menstrual cycle; (2) the possibility of future pregnancies where there is a risk of the tumour regrowing causing symptoms with greater severity and possibly at an earlier time. However, there have not been any studies that quantify the probability of this happening and the outcomes of such patients. In the absence of evidence, careful shared decision-making between the clinical team and the patient is appropriate.

## 4. Conclusion

Meningiomas are known to grow in size during pregnancy and can be misdiagnosed as other more common pregnancy related conditions. The tumour should always be managed conservatively during pregnancy, unless there is rapid deteriorating neurology. Postpartum, the decision to excise the tumour should be made after careful discussion between the patient and the clinical team.
